# Chicago Medical Society

**Published:** 1879-11

**Authors:** 


### CHICAGO MEDICAL SOCIETY.

*Laceration of the Cervix Uteri.*—At a meeting of the Chicago Medical Society, held July 7th, Dr. A. Reeves Jackson read a paper on " Laceration of the Cervix Uteri," of which the following is an abstract:

The first recognition of this lesion as a factor in the causation of uterine disease was made by Dr. A. K. Gardner, in 1856, but to Dr. Thomas Addis is due the credit of calling attention to the frequency and pathological importance of the injury, and above all, of showing how to recognize and cure it. Indeed, Dr. Emmet has so identified himself with the subject, and his writings have been so full and complete, that but little has been left for subsequent writers to do, save add to his facts or corroborate his views. Though we cannot all be discoverers of important truths, we may aid in their dissemination, and to accomplish this was Dr. Jackson's earnest desire, as he believed that the profession at large does not properly appreciate the importance of the subject.

He began by calling attention to the fact, not generally recognized, that injuries to the uterine cervix during the process of parturition, are of extremely frequent occurrence; that they are of great and enduring pathological moment; that they are frequently the undiscovered cause of many of the derangements of health in women which have been, and still are, habitually attributed to other sources and conditions; that the venerable myth "ulceration," and its Fidus Achates, enlargement of the cervix, are so rare (except when the seat of malignant disease) that we might reach ripe old age without seeing either; and that these terms in such common use may and should be in almost every instance "laceration of the cervix."

When these truths—for such he held them to be—are known and accepted, there will not be so many nervous, weak-backed, tired out women as now crowd our waiting rooms; and we shall hear less of the uselessness of pessaries for "falling" wombs, and less of amputation for enlarged ones, less of failures to cure "the whites;" and see less pallid patients wandering hopelessly from one doctor to another, only to have repeated by each in turn the "burning off" of ulcers!

Dr. Jackson cited interesting instances from his own practice, pointed out how easily errors may be made or avoided; quoted twenty-three cases in which he had operated, nearly all of which were attended with the most satisfactory results, and some without even serious symptoms. He then proceeded to discuss the subject in a systematic manner under the heads of "frequency," "causes," "varieties," "progress," "symptoms," "diagnosis," and "treatment," and said in conclusion, that the operation was one of the most successful with which he was acquainted, and when its simplicity and freedom from danger are considered in relation to the amount of benefit which it is capable of conferring, it may be justly regarded as one of the most useful and important in uterine surgery.

In the discussion which followed the paper, among other speakers, Dr. John A. Bartlett, related a case in the practice of Dr. Mary Thompson. A woman suffering from laceration of the cervix uteri became the subject of epileptic seizures, and finally had in one day six or eight attacks. Dr. T. operated in the usual way, and the patient recovered to the extent that she had only had one seizure in three months past.

Dr. Bartlett also referred to a paper which he had read formerly "On the Anatomy of the Cervix Uteri, before, during, and after labor," and wished to call attention to the facts therein pointed out as having a new interest in connection with the subject under discussion.

The majority of recent writers affirm that, until shortly prior to confinement, the cervix remains entirely undeveloped; and that the tip of the finger passed along the cavity of the neck may reach the *os internum;* that during the process of labor the uterus and vagina form a continuous canal, and that after labor the strongly 'pursed' orifice, through which the fingers enter the womb, is the *os externum.* Dr. Bartlett in the paper referred to, maintained that before labor the cervix had greatly expanded, so that even that portion of it below the vaginal attachment measured several inches in width; that the contracted portion felt by the writers alluded to *below* the vaginal attachment, and yet located, in their imagination, *above* it, was not, as they supposed, the *os internum*, but was that segment of the cervix which at the time of examination was the line of demarkation between the expanded and yet unexpanded portion of the neck.

He had also stated that the infra-vaginal portion of the cervix was *not* obliterated at the time of the passage of the child, but remained as a narrow band jutting into the parturient canal. And finally, he claimed that after labor, the firmly contracted orifice generally alluded to as "the *os*" is the *os internum*, while the infra-vaginal portion of the cervix floats in the vagina as a flabby ring.

It was, therefore, the fact of the existence of this ring which he wished to introduce in this connection. For while no one could conceive of those conditions which are now recognized as cervical lacerations as being the outcome of the slight, superficial, 'purse-mouth' flutings which may be sometimes felt in the margin of the *os* after labor; yet, given an actual, substantial fleshy ring, through which is to be propelled a fœtus, it is easy to conceive of the cause, the time, the anatomy, the means of diagnosis, and even the proper plan of treatment of those lesions of the uterine neck just now of such paramount interest to the gynæcologist.—*Southern Clinic.*

